# Anti-Inflammatory Activities of an Extract of In Vitro Grown Adventitious Shoots of *Toona sinensis* in LPS-Treated RAW264.7 and *Propionibacterium acnes*-Treated HaCaT Cells

**DOI:** 10.3390/plants9121701

**Published:** 2020-12-03

**Authors:** Hyeon-Ji Lim, In-Sun Park, Eun Yee Jie, Woo Seok Ahn, Sang-Jun Kim, Seung-Il Jeong, Kang-Yeol Yu, Suk Weon Kim, Chan-Hun Jung

**Affiliations:** 1 Jeonju AgroBio-Materials Institute, Jeonju-si, Jeollabuk-do 54810, Korea; lhj0923@jami.re.kr (H.-J.L.); witwit58@jami.re.kr (I.-S.P.); process95@jami.re.kr (S.-J.K.); sijeong@jami.re.kr (S.-I.J.); kangyu@jami.re.kr (K.-Y.Y.); 2 Biological Resource Center, Korea Research Institute of Bioscience & Biotechnology, Jeongeup-si, Jeollabuk-do 56212, Korea; jeannie@kribb.re.kr (E.Y.J.); dntjr0412@kribb.re.kr (W.S.A.)

**Keywords:** *Toona sinensis*, adventitious shoots, antioxidation, antibacterial activity, anti-inflammation, *P. acnes*-induced inflammatory disease

## Abstract

*Toona sinensis* has been traditionally used to treat dysentery, enteritis, flatulence, and itchiness. However, the existence of anti-inflammatory effects of *T. sinensis* on *Propionibacterium acnes*-induced skin disease is unknown. In vitro cultures of plant cells and tissues produced under controlled conditions offer a continuous production platform for plant natural products including pigments and anti-inflammatory agents. In this study, we determine the anti-inflammatory activities of an extract of in vitro grown adventitious shoots of *T. sinensis* on *P. acnes*, the etiologic agent of skin inflammation. The extract of *T. sinensis* showed antioxidant and anti-inflammatory activity in LPS-treated RAW264.7 cells. It also had antibacterial activity and anti-inflammatory effects on *P. acnes*-treated HaCaT cells. In addition, these effects were regulated by suppression of the mitogen-activated protein kinase (MAPK) pathways. These results suggesting the potential application of adventitious shoots of *T. sinensis* grown with an in vitro proliferation system as a medicine for treating *P. acnes*-induced inflammatory skin disease.

## 1. Introduction

Acne is one of the most common skin diseases globally, affecting approximately 650 million adolescents and adults [1,2]. Acne is a chronic inflammatory disease which causes skin redness, itching, and inflammation [3]. Although the exact causes of acne remain unclear, environmental factors such as heredity, diet, stress, hormonal imbalance, and a narrow equilibrium between *Propioninbacterium acnes* (*P. acnes*) and other skin flora might play important roles in acne onset [4,5,6]. Among these factors, *P. acnes*, a Gram-positive anaerobic bacterium, has been reported to be a major factor in acne inflammatory reaction [7]. *P. acnes* may sequentially trigger toll-like receptors 2 (TLR2) and activate mitogen-activated protein kinase (MAPKs) and transcription factor nuclear factor-κB (NF-κB) signaling pathways, leading to the production of pro-inflammatory cytokines such as interleukin (IL)-1β, IL-6, IL-8, and tumor necrosis factor (TNF)-α in monocytes and keratinocytes [7,8,9]. In addition, enhanced expression of IL-8 and IL-12 due to the activation of TLR-2 can stimulate hyperkeratinization, inflammation and oxidative stress [10,11].

Plant cell or tissue cultures are important tools for the genetic transformation of useful plant genotypes [12] and the continuous production of plant-derived metabolites with commercial value [13,14]. Plant cell or tissue cultures capable of producing useful metabolites offer a number of advantages over traditional field cultivation, including independence from geographical, seasonal, and environmental variations, uninterrupted production in uniform quality and yield, no need for pesticide or herbicide application, and comparatively short growth cycles [15,16]. Thus, in vitro cultures of plant cells and tissues under controlled conditions offer a continuous production platform for plant natural products including pigments and anti-inflammatory agents [17,18,19].

*Toona sinensis* (Juss.) M. Roem. (syn. *Cedrela sinesis* Juss.), commonly known as Chinese toon, mahogany, and cedar, belonging to family Meliaceae [20]. *T. sinensis* (TS), has been cultivated in Asia, including China, for a long time, and is widely used in China and Korea as traditional medicine and as a food [21]. It has various pharmacological activities, including antioxidant, anti-inflammatory, anticancer, anti-diabetic, anticoagulant, anti-gout, hepatoprotective, antibacterial, and antiviral effects [22,23]. Studies on the chemical constituents of TS have shown the presence of terpenoids, phenylpropanoids, flavonoids, and anthraquinones [24]. Among the chemical constituents of TS, terpenoids have a broad spectrum of various pharmacological functions, such as anti-inflammatory, anti-bacterial, antiviral, anticancer, and hepatoprotective activities [25,26]. Other chemical constituents of TS including phenylpropanoids, flavonoids, and anthraquinones also exhibit antioxidant activities [24]. However, the antibacterial and anti-inflammatory effects of TS on *P. acnes*-induced inflammation have not been reported to date. Therefore, the objective of this study was to evaluate the antibacterial and anti-inflammatory activities of TS extracts prepared from the dried adventitious shoots of TS and in LPS-induced RAW264.7 and *P. acnes*-treated HaCaT cells.

## 2. Materials and Methods

### 2.1. Induction and Proliferation of Adventitious Shoots of T. sinensis

**Figure 1:**
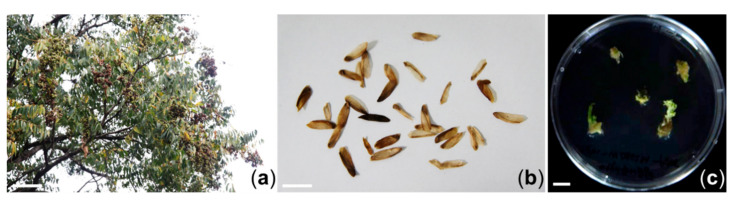
Establishment of an in vitro proliferation system from hypocotyl explants of *T. sinensis*: (**a**) Field grown plant of *T. sinensis*; (**b**) Mature seeds of *T. sinensis*; (**c**) In vitro grown adventitious shoots of *T. sinensis*. Scale bar, 1 m in (**a**) and 1 cm in (**b**,**c**).

Seeds of *Toona sinensis* (Juss.) M. Roem were collected from Jeongeup-si, Jeollabuk-do province, Korea in 2016 (Figure 1a,b). Seeds were sterilized in 70% (*v/v*) EtOH for 0.5 min and soaked in 0.8% sodium hypochlorite (NaOCl) solution for 20 min. After surface sterilization, seeds were rinsed thoroughly with sterilized and distilled water. To remove the remaining NaOCl solution, these washing processes were repeated three times. After washing, any remaining moisture was removed with sterilized filter paper (Advantec, Tokyo, Japan; 70 mm). The seeds were transferred to MS [19] basal medium supplemented with 0.4 mg/L thiamine-HCl, 100 mg/L myo-inositol, 3% (*w/v*) sucrose, and 0.4% (*w/v*) Gelrite. The pH of the medium was adjusted to 5.8 with 1N NaOH. Cultures were maintained at 25 °C in the dark for seed germination.

To induce adventitious shoots, hypocotyl explants from two week-old seedlings were collected and cut into small segments (approximately 5 mm in length). Hypocotyl explants were place onto MS medium supplemented with 2 mg/L BA (benzyl aminopurine), 0.1 mg/L NAA (α-naphthaleneacetic acid), 0.4 mg/L thiamine-HCl, 100 mg/L myo-inositol, 3% (*w/v*) sucrose, and 0.4% (*w/v*) Gelrite (MS2B01N). Each treatment of ten explants had three replicates. All cultures were maintained at 25 °C with light (approximately 30 µmol/m^2^s, light/dark regime of 16/8 h) unless otherwise mentioned.

### 2.2. Preparation of TS Extract

After four weeks of incubation, rapidly growing adventitious shoots derived from hypocotyl explants of *T. sinensis* were transferred to fresh MS2B01N medium and incubated at 25 °C with light for four weeks. After four weeks of incubation, rapidly growing adventitious shoots were collected, freeze-dried, ground into a fine powder, dissolved in dimethyl sulfoxide (DMSO), and sonicated for 1 h. After centrifugation at 12,000 rpm for 5 min, the supernatant (TS extract) was used for this study.

### 2.3. DPPH Scavenging Activity Assay

DPPH (1,1-diphenyl-2-picrylhydrazyl) scavenging activity assay was performed as described previously [19]. In brief, 100 μL of the TS extract at different concentrations (50, 100, 250, and 500 μg/mL) was mixed with 100 μL of freshly prepared 0.1 mM DPPH in ethanol and incubated at 37 °C in the dark for 30 min. The absorbance of the mixture was measured at 517 nm with a microplate reader (Thermo Scientific, Waltham, MA, USA). The percentage inhibition of DPPH radical scavenging activity was calculated with the following formula:

 DPPH scavenging activity (%) = 1 − (Absorbance of control − Absorbance of sample)/Absorbance of control × 100%(1)

### 2.4. Superoxide Dismutase (SOD) Activity Assay

A superoxide dismutase (SOD) activity assay was performed using an SOD assay kit (Sigma Aldrich, St. Louis, MO, USA). In brief, 20 μL of the TS extract at different concentrations (50, 100, 250, and 500 μg/mL) was mixed with 220 μL of the reaction mixture in the SOD kit and incubated at 37 °C for 20 min. The absorbance of the mixture was measured at 450 nm with a microplate reader (Thermo Scientific, Waltham, MA, USA). The SOD activity (percentage of inhibition of WST-1 reduction) was determined with the following formula:

SOD inhibition activity (%) =   1 − (Absorbance of control − Absorbance of sample)/Absorbance of control × 100%(2)

### 2.5. Cell Cultures

Murine macrophage cell line RAW264.7 was purchased from Korean Cell Line Bank (Seoul, Korea). Human keratinocyte cell line HaCaT was purchased from CLS Cell Lines Service (Eppelheim, Germany). RAW264.7 and HaCaT cells were cultured in DMEM (WELGENE, Seoul, Korea) supplemented with 10% fetal bovine serum (Hyclone, Logan, UT, USA) and incubated at 37 °C in an incubator with 5% CO_2_.

### 2.6. Antibacterial Assay

Antibacterial activity was determined using an agar disc diffusion method. *P. acnes* was purchased from the American Type Culture Collection (Manassas, VA, USA) and cultured with Brain Heart Infusion (BHI) agar (BD Biosciences, San Jose, CA, USA) in anaerobic condition at 37 °C. Sterile filter paper discs (8 mm) were treated with 1, 2, and 3 mg/disc of TS extract or DMSO and placed on BHI agar plate spread with *P. acnes*. Clear zones of growth inhibition around discs were measured after 48 h of incubation at 37 °C.

### 2.7. Cell Viability Assay

RAW264.7 cells and HaCaT cells seeded into 96-well plates at a density of 5 × 10^3^ cells/well and incubated at 37 °C for 24 h. The cultured cells were then treated with various concentrations (50, 100, 250, and 500 μg/mL) of TS extract for 24 h. After adding 5 mg/mL of MTT reagent (Sigma Aldrich, St. Louis, MO, USA) to each well, cells were incubated for 4 h. The supernatant was removed and the formazan crystal in each well was solubilized with 100 μL DMSO. The absorbance was then measured at 570 nm with a microplate reader (Thermo Scientific, Waltham, MA, USA).

### 2.8. Measurement of NO and PGE_2_

RAW264.7 cells were seeded into 24-well plates at a density of 5 × 10^4^ cell/well. These cells were pretreated with various concentrations (50, 100, 250, and 500 μg/mL) of TS extract for 1 h. Cells were then treated with 1 μg/mL of LPS. After 24h, the supernatant in each well was collected for nitric oxide (NO) and PGE_2_ determination. Concentrations of NO and PGE_2_ in supernatants were measured with Griess Reagent (Promega, Madison, WI, USA) and ELISA kits (R&D Systems, Minneapolis, MN, USA) according to each manufacturer’s instructions.

### 2.9. Enzyme-Linked Immunosorbent Assay (ELISA)

RAW264.7 cells were seeded into 24-well plates at a density of 5 × 10^4^ cell/well. These cells were pretreated with various concentrations (50, 100, 250, and 500 μg/mL) of TS extract for 1 h. Cells were then treated with 1 μg/mL of LPS. After 24 h, the supernatant was collected from each well for ELISA mouse IL-6 cytokine (BD Biosciences).

HaCaT cells were seeded into 24-well plates at a density of 5 × 10^4^ cell/well. Cells were then pretreated with various concentrations (50, 100, 250, and 500 μg/mL) of TS extract for 4 h. After that, they were treated with *P. acnes* for 18 h. The supernatant was then collected from each well for ELISA measurement of IL-6 and IL-8 cytokines (BD Biosciences). In Brief, 96-well plates were coated with 100 μL of capture antibodies and incubated overnight at 4 °C. Plates were then washed three times with washing buffer (0.05% Tween in PBS) and blocked with an assay diluent (10% FBS in PBS) at room temperature for 1 h. After the medium was aspirated, 100 μL of each standard or sample was added to each well for 2 h. Plates were then washed again and 100 μL of detection antibody solution was added to each well for 2 h. Accordingly, 100 μL of TMB substrate solution was added to each well and incubated at room temperature for 30 min in the dark. The absorbance was measured at 450 nm with a microplate reader (Thermo Scientific, Waltham, MA, USA).

### 2.10. Real-Time RT-PCR

Total RNA was extracted using RNA extraction kit (GeneAll, Seoul, Korea) according to the manufacturer’s protocols. Total RNA was reversed-transcribed to cDNA with cDNA synthesis Kit (BioFACT, Seoul, Korea). Real-time RT-PCR was performed using SYBR Green qPCR Master Mix (BioFACT, Seoul, Korea) and a Real-time PCR system (Bio-Rad, Hercules, CA, USA). Primer sequences are shown in Table 1. Relative amounts of mRNAs were calculated based on the threshold cycle number using β-actin as an endogenous control. All experiments were performed in triplicate and values were averaged.

### 2.11. Western Blot Analyses

RAW264.7 and HaCaT cells were seeded into six-well plates at a density of 5 × 10^5^ cell/well. These cells were pretreated with various concentrations (50, 100, 250, and 500 μg/mL) of TS extract for 2 h and then treated with LPS (1 μg/mL) for 24 h or *P. acnes* for 2 h. RAW264.7 cells were harvested with PBS and lysed using lysis buffer (Thermo Scientific, Waltham, MA, USA) for 1 h. Proteins in cell lysates were separated by 10% sodium dodecyl sulfate (SDS)-polyacrylamide gel electrophoresis (SDS-PAGE) and then transferred to PVDF membranes (Merck, Darmstadt, Germany). Membranes were blocked with 5% BSA for 2 h and incubated at 4 °C overnight with primary antibodies: anti-iNOS, anti-COX-2, anti-p38, antiphospho-p38, antiphospho-ERK, antiphospho-JNK, and anti-β-actin antibodies from Cell Signaling Technology (Danver, MA, USA), and anti-ERK, anti-JNK, and anti-GAPDH from Santa Cruz Biotechnology (Santa Cruz, CA, USA). Blots were washed with TBST (Tris-buffered saline, 0.1% Tween 20) for 1 h and incubated with secondary antibodies for 30 min. Protein bands were detected using ECL western blotting detection reagents (GE healthcare, Buckinghamshire, UK) and an Amersham Imager 600 (GE healthcare, Buckinghamshire, UK).

### 2.12. Statistical Analysis

All experiments were repeated at least three times. Statistical significance was determined using Student’s *t*-test or one-way analysis of variance (GraphPad Software, La Jolla, CA, USA).

## 3. Results and Discussion

### 3.1. In Vitro Proliferation of Adventitious Shoots of T. sinensis

Adventitious shoots were successfully induced from hypocotyl explants of *T. sinensis* when they were cultured on MS2B01N medium after four weeks of incubation. These shoots were transferred to fresh MS2B01N medium and subcultured at four-week intervals (Figure 1c). After subculture, proliferated calluses were collected carefully and freeze-dried to determine antioxidant, tyrosinase inhibition, and antibacterial activities. A HPLC-DAD analysis was performed on a methanol extract to confirm the quality of this plant material (Supplementary Materials Figure S1).

### 3.2. Anntioxidant Effects of TS Extract

**Figure 2:**
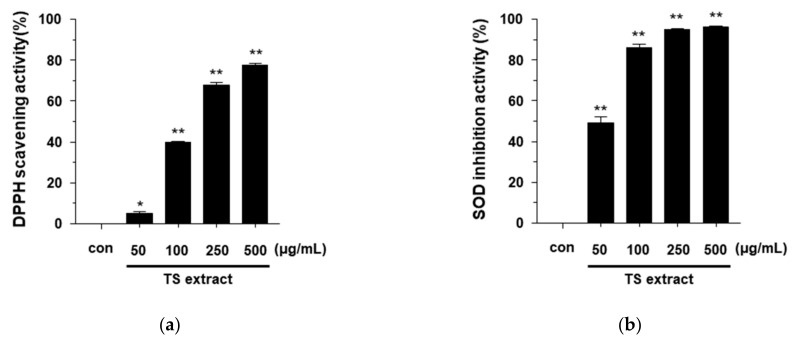
Antioxidant activity of TS extract. (**a**) DPPH scavenging activity of TS extract (50, 100, 250, and 500 μg/mL) was measured by DPPH assay; (**b**) SOD inhibition activity of TS extract (50, 100, 250, and 500 μg/mL) was determined by SOD assay kit. Ascorbic acid was used as a positive control. Values are expressed as mean ± SD of three independent experiments. * *p* < 0.05; ** *p* < 0.001 vs. the control.

*T. sinensis* is known to have antibacterial and anti-inflammatory activities, and commonly used as a natural herbal medicine for treating dysentery, enteritis, and itchiness [3]. However, the anti-inflammatory effects of TS on *P. acnes*-induced inflammation disease remain unknown. To investigate the bioactivities of TS on *P. acnes*-induced inflammation, we first prepared a TS extract from dried adventitious shoots of TS. As acne lesions are known to be associated with high reactive oxygen species (ROS) [27,28], we performed DPPH and SOD assays to measure the antioxidant activity of TS extract. Ascorbic acid was used as a positive control (Supplementary Materials Figure S2). Figure 2a shows the DPPH radical scavenging activity of TS extract in a dose-dependent manner. As shown in Figure 2b, the antioxidant effect was confirmed using an SOD assay. The IC_50_ values were 177.1 ± 5.18 μg/mL for TS extract and 5.0 ± 0.26 μg/mL for ascorbic acid in the DPPH methods, and 56.5 ± 2.97 μg/mL and 14.3 ± 0.22 μg/mL in the SOD assay, respectively. This finding indicated that the TS extract possessed significant antioxidant activity.

### 3.3. Cytotoxicity and Anti-inflammatiory Effects of TS Extract on RAW264.7 Cells

**Figure 3:**
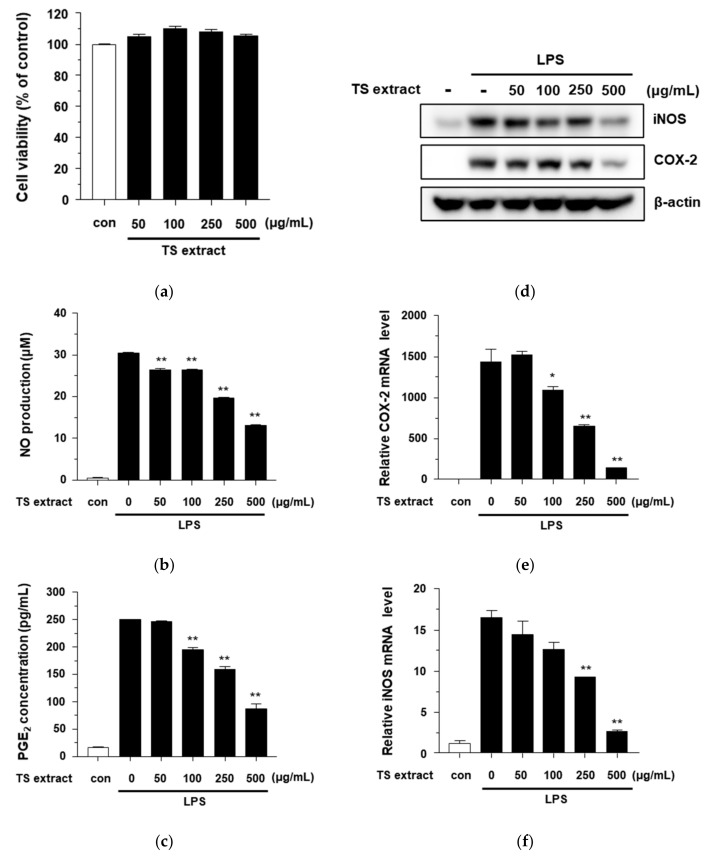
Anti-inflammatory effects of TS extract in LPS-treated RAW264.7 cells. (**a**) Cells were treated without or with the indicated concentration of TS extract and cell viability was determined by MTT assay; (**b**) Cells were pretreated with indicated concentration of TS extract for 1 h and then incubated with 1 ug/mL of LPS for 24 h. The level of nitrite (NO) was detected using a Griess reaction assay; (**c**) The level of PGE_2_ was determined using ELISA kit; (**d**) Protein levels of COX-2 and iNOS were determined by western blot; (**e**,**f**) Relative mRNA levels of COX-2 and iNOS were determined by real-time PCR. Values are expressed as mean ± SD of three independent experiments; * *p* < 0.05; ** *p* < 0.001 vs. LPS alone.

The cytotoxicity of TS extract to RAW264.7 cells was analyzed by exposing cells to various concentrations (50, 100, 250, and 500 μg/mL) of the extract for 24 h. As shown in Figure 3a, the TS extract did not show any significant cytotoxic effects compared to the control. To test the anti-inflammatory effects of the TS extract under a noncytotoxic condition, the inhibitory activity of the extract against LPS-induced NO production in RAW264.7 cells was first investigated. A nitric oxide synthesis inhibitor, L-NMMA, was used as a positive control (Supplementary Materials Figure S3). As shown in Figure 3b, the TS extract significantly decreased LPS-induced NO production. The TS extract and L-NMMA exhibited NO inhibitory activities with IC_50_ values of 400.37 ± 3.72 μg/mL and 83.42 ± 2.67 μM, respectively (Figure 3b and Figure S3). Next, to confirm anti-inflammatory effects of the TS extract, the inhibitory activity of the extract against LPS-induced PGE_2_ production in RAW264.7 cells was investigated. RAW264.7 cells were pretreated with TS extract at various concentrations (0, 50, 100, 250, and 500 μg/mL) for 1 h and then treated with 1 μg/mL of LPS for 24 h. Levels of PGE_2_ in culture supernatants were determined with Griess assay and an ELISA kit. As shown in Figure 3c, the TS extract significantly reduced levels of PGE_2_ in LPS-treated RAW274.7 cells in a dose-dependent manner. These results indicate that TS extract has anti-inflammatory effects by inhibiting NO and PGE_2_ production. It has been reported that NO and PGE_2_ are synthesized by iNOS and COX-2 in inflammatory reactions [29]. Therefore, the question of whether the inhibitory effects of the TS extract on NO and PGE_2_ production were related to the regulation of expression of their synthesis enzymes iNOS and COX-2, respectively, was investigated. As shown in Figure 3d, the TS extract significantly reduced protein levels of iNOS and COX-2 in LPS-treated RAW264.7 cells in a dose-dependent manner. We also measured the mRNA levels of iNOS and COX-2 by real-time PCR. The results showed that the TS extract significantly inhibited the mRNA levels of COX-2 and iNOS in a dose-dependent manner (Figure 3e,f). Moreover, the TS extract significantly reduced the secretion of IL-6 in LPS-treated RAW264.7 cells (Supplementary Materials Figure S4). These results suggest that TS extract can reduce NO and PGE_2_ production by suppressing the mRNA levels of iNOS and COX-2.

### 3.4. Antibacterial Activity of TS Extract Against P. acnes

**Figure 4:**
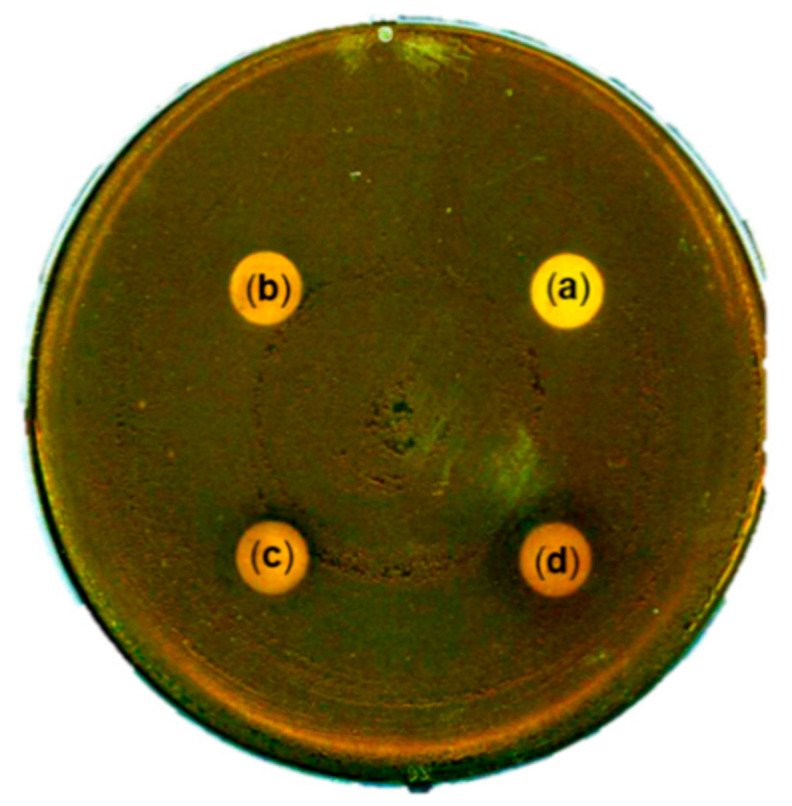
Antibacterial activity of TS extract. (**a**) DMSO; (**b**) 1 mg/disc of TS extract; (**c**) 2 mg/disc of TS extract; (**d**) 3 mg/disc of TS extract.

Inflammatory response, including the pro-inflammatory mediators (iNOS and COX-2) and cytokines (IL-1β, IL-6, and IL-8), is associated with inflammatory skin diseases such as acne [7,8,9,30]. The TS extract showed anti-inflammatory effects by suppressing the pro-inflammatory mediators (iNOS and COX-2) and cytokine (IL-6) production in LPS-treated RAW264.7 cells (Figure 3 and Figure S4). To determine the antibacterial activity of TS extract against *P. acnes*, agar disc diffusion assay was performed using DMSO as a negative control. As shown in Figure 4, a TS extract at concentrations of 2 mg/disc and 3 mg/disc resulted in clear zones of 1 mm and 1.4 mm in diameter, respectively. However, a lower concentration (1 mg/disc) of TS extract had no antibacterial activity against *P. acnes*. This finding indicates that TS extract could inhibit the growth of *P. acnes* at concentrations of 2 mg/disc or higher.

### 3.5. Anti-Inflammatory Effects of TS Extract in P. acnes-treated HaCaT Cells

**Figure 5:**
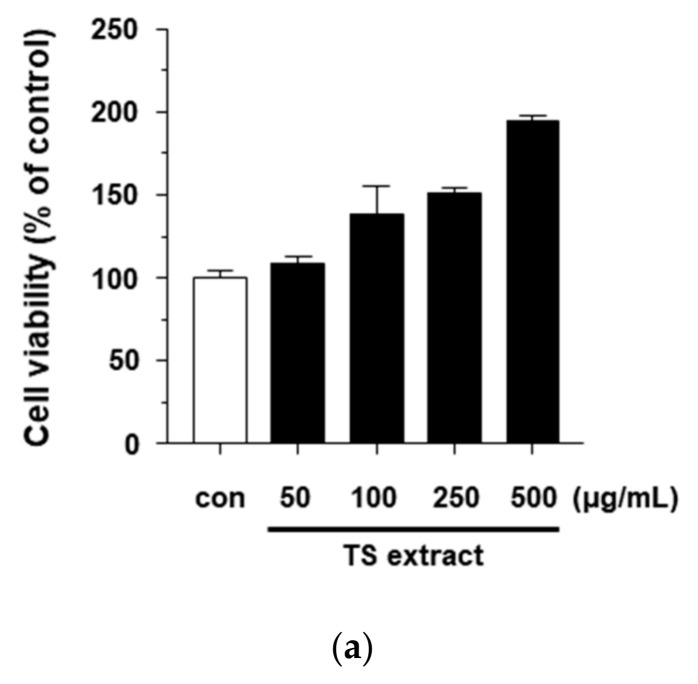

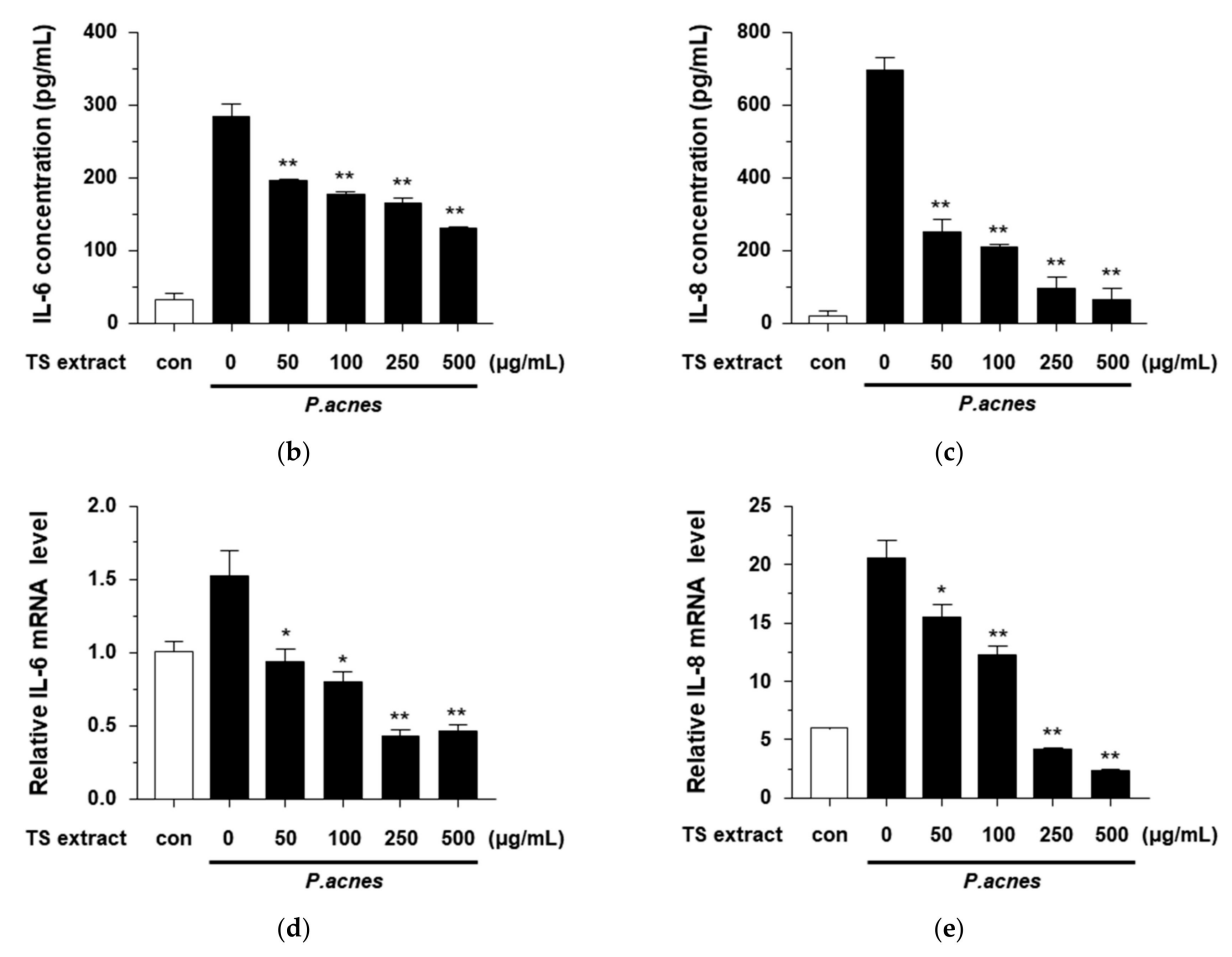
Anti-inflammatory effects of TS extract in *P. acnes*-treated HaCaT cells. (**a**) Cells were treated without or with indicated concentrations of TS extract and cell viability was determined by MTT assay; (**b**) Cells were pretreated with indicated concentrations of TS extract for 4 h and then treated with *P. acnes* for 18 h. Secretion level of IL-6 was determined by ELISA kit; (**c**) Secretion level of IL-8 was determined by ELISA kit; (**d**,**e**) Relative mRNA levels of IL-6 and IL-8 were determined by real-time PCR. Values are expressed as mean ± SD of three independent experiments; * *p* < 0.05; ** *p* < 0.001 vs. *P. acnes* alone.

The cytotoxicity of the TS extract to HaCaT cells was analyzed by exposing cells to various concentrations (50, 100, 250, and 500 μg/mL) of the extract. As shown in Figure 5a, the TS extract did not show any cytotoxic effect. To determine the anti-inflammatory effects of the TS extract against *P. acnes*-treated HaCaT cells, the inhibitory activities on *P. acnes*-induced inflammatory cytokine secretion in HaCaT cells were investigated. HaCaT cells were pretreated with various concentrations (0, 50, 100, 250, and 500 μg/mL) of TS extract for 4 h, and then treated with *P. acnes* for 18 h. As shown in Figure 5b,c, the TS extract significantly suppressed the secretion of IL-6 and IL-8 in HaCaT cells. We also determined the mRNA levels of IL-6 and IL-8 in *P. acnes*-treated HaCaT cells. The results showed that the TS extract significantly inhibited the mRNA levels of IL-6 and IL-8 in a dose-dependent manner (Figure 5d,e). These results suggest that TS extract can inhibit the production of pro-cytokines IL-6 and IL-8 by reducing their mRNA levels in *P. acnes*-treated HaCaT cells.

### 3.6. Regulatory Effects of TS Extract on Activated MAPK Signaling Pathway in P. acnes-Treated HaCaT Cells

**Figure 6:**
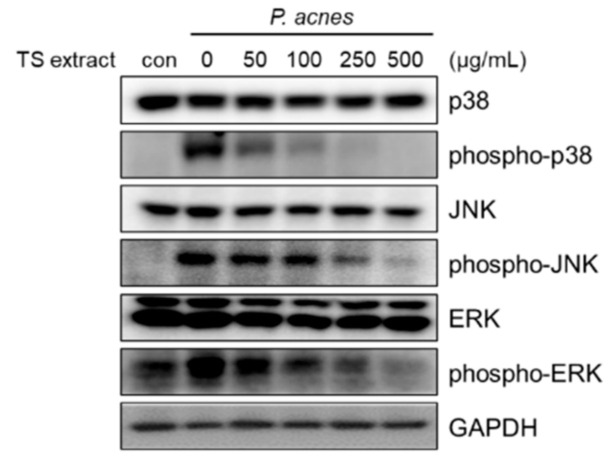
Effect of TS extract on the MAPK signaling pathway in *P. acnes*-treated HaCaT cells. Cells were pretreated with indicated concentrations of TS extract for 2 h and then treated with *P. acnes* for 2 h. The levels of p38, phospho-p38, JNK, phospho-JNK, ERK, and phospho-ERK were compared by Western blotting. GAPDH was used as a loading control.

It has been reported that *P. acnes* can induce inflammatory response via the MAPK signaling pathway [7,8,9]. Therefore, the question of whether the anti-inflammatory effects of the TS extract were regulated by the MAPK signaling pathway was investigated. As shown in Figure 6, the expression levels of phosphorylated p38, JNK, and ERK following treatment of *P. acnes* were increased. However, the expression levels of phosphorylated p38, JNK, and ERK decreased significantly by treatment with the TS extract. These results suggest that TS extract can inhibit *P. acnes*-induced inflammatory response by regulation of the MAPK signaling pathway in HaCaT cells.

## 4. Conclusions

We established an in vitro proliferation system for adventitious shoots of *T. sinensis* and demonstrated that a TS extract had antibacterial and anti-inflammatory activities against *P. acnes*-induced inflammation by inhibiting the release of pro-inflammatory cytokines via suppression of MAPK signaling pathways. These findings suggest that a TS extract might have applications as a medicine for treating *P. acnes*-induced inflammatory diseases. However, since these effects were observed at high concentrations, further in vitro and in vivo percutaneous absorption studies are required for applications to treat *P. acnes*-induced inflammatory diseases.

## Figures and Tables

**Table 1:** Primer sequences used for real-time PCR.

Gene		Sequence (5’ to 3’)
COX-2	Forward	CTCTACAACAACTCCATCCT
(mouse)	Reverse	ATTCTGCAGCCATTTCCTTC
iNOS	Forward	CGAAACGCTTCACTTCCAA
(mouse)	Reverse	TGAGCCTATATTGCTGTGGCT
β-actin	Forward	CGGTTCCGATGCCCTGAGGCTCTT
(mouse)	Reverse	CGTCACACTTCATGATGGAATTGA
IL-6	Forward	AGCCACTCACCTCTTCAGAAC
(human)	Reverse	GCCTCTTTGCTGCTTTCACAC
IL-8	Forward	CTGATTTCTGCAGCTCTGTG
(human)	Reverse	GGGTGGAAAGGTTTGGAGTATG
GAPDH	Forward	TGCACCACCAACTGCTTAGC
(human)	Reverse	GGCATGGACTGTGGTCATGAG

## Supplementary Materials

The following are available online at https://www.mdpi.com/2223-7747/9/12/1701/s1, Figure S1: HPLC chromatograms of in vitro grown adventitious shoots of *T. sinensis*., Figure S2: Antioxidant activity of ascorbic acid., Figure S3: Anti-inflammatory effects of NG-nitro-L-arginine methyester (L-NMME) in LPS-treated RAW264.7 cells., Figure S4: Anti-inflammatory effects of TS extract in LPS-treated RAW264.7 cells.
