# Supplementary material for: Anti-Inflammatory Activities of an Extract of In Vitro Grown Adventitious Shoots of Toona sinensis in LPS-Treated RAW264.7 and Propionibacterium acnes-Treated HaCaT Cells
Source: Plants (Basel). 2020 Dec 3;9(12):1701. doi: 10.3390/plants9121701 (PMC7761664; doi:10.3390/plants9121701)
Supplement: Supplementary file 1 [file plants-09-01701-s001.pdf]

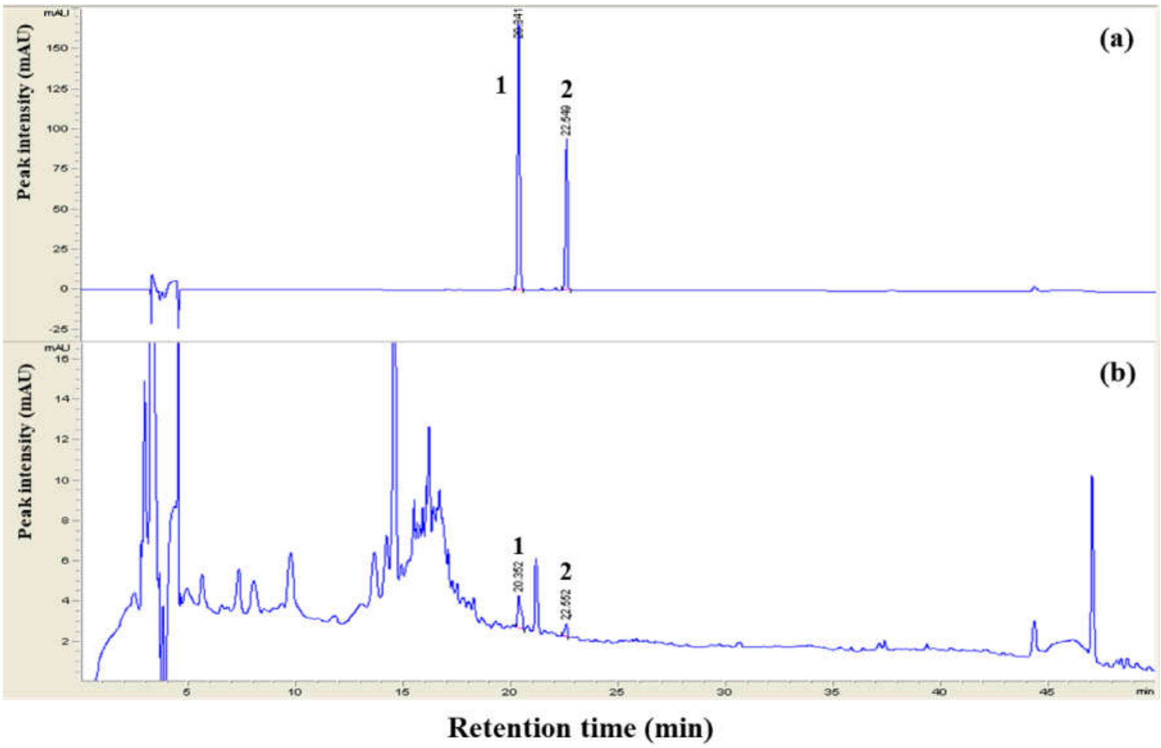

Figure S1. HPLC chromatograms of *in vitro* grown adventitious shoots of *T. sinensis*. (**a**) standard solution: 1, quercitrin; 2, afzelin. (**b**) methanol extract. Quercitrin (1) and afzelin (2) as the marker were eluted with ODS analytical column and detected at UV 254 nm.

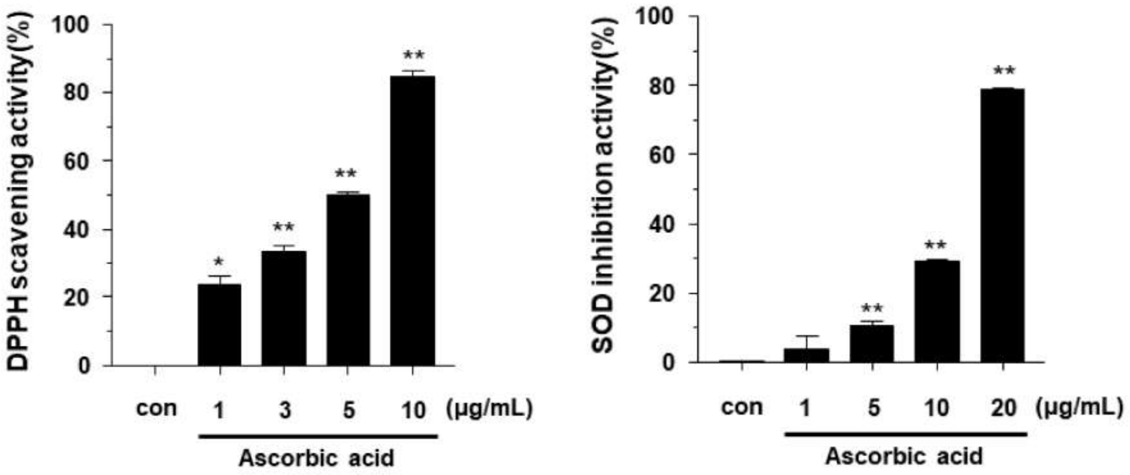

(**a**) (**b**)

Figure S2. Antioxidant activity of ascorbic acid. (**a**) DPPH scavenging activity of ascorbic acid (1, 3, 5, and 10 μg/ml) was measured by DPPH assay; (**b**) SOD inhibition activity of ascorbic acid (1, 5, 10, and 20 μg/ml) was determined by SOD assay kit. Values are expressed as mean ± SD of three independent experiments. *, $p < 0.05$; **, $p < 0.001$ versus the control.

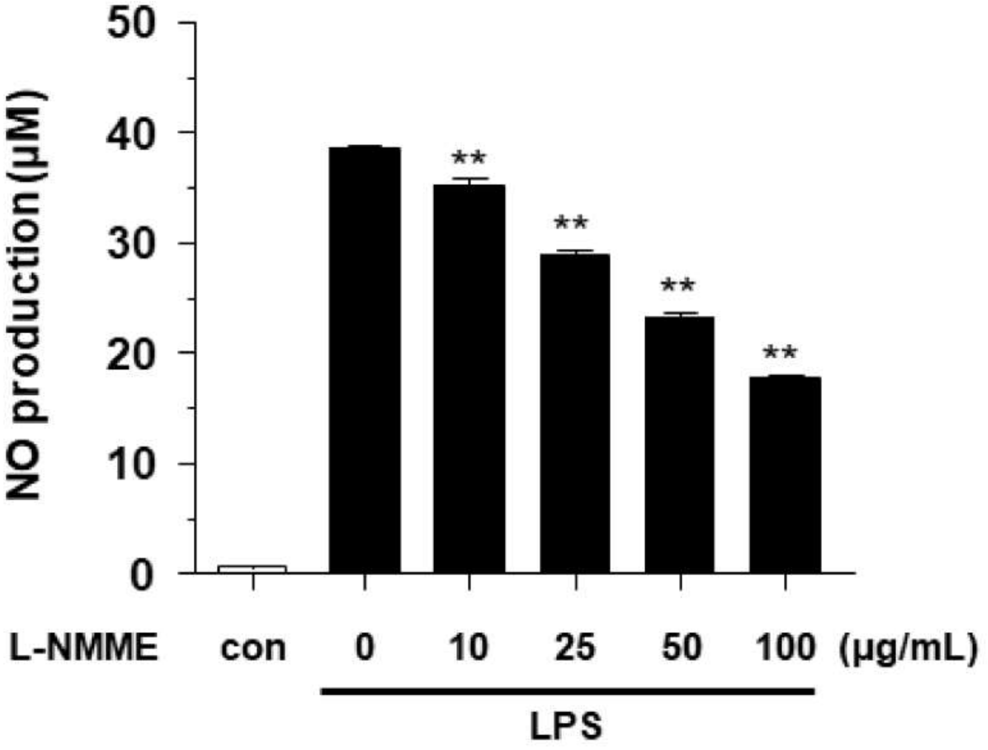

Figure S3. Anti-inflammatory effects of NG-nitro-L-arginine methylester (L-NMME) in LPS-treated RAW264.7 cells. Values are expressed as mean ± SD of three independent experiments. **, $p < 0.001$ versus the control.

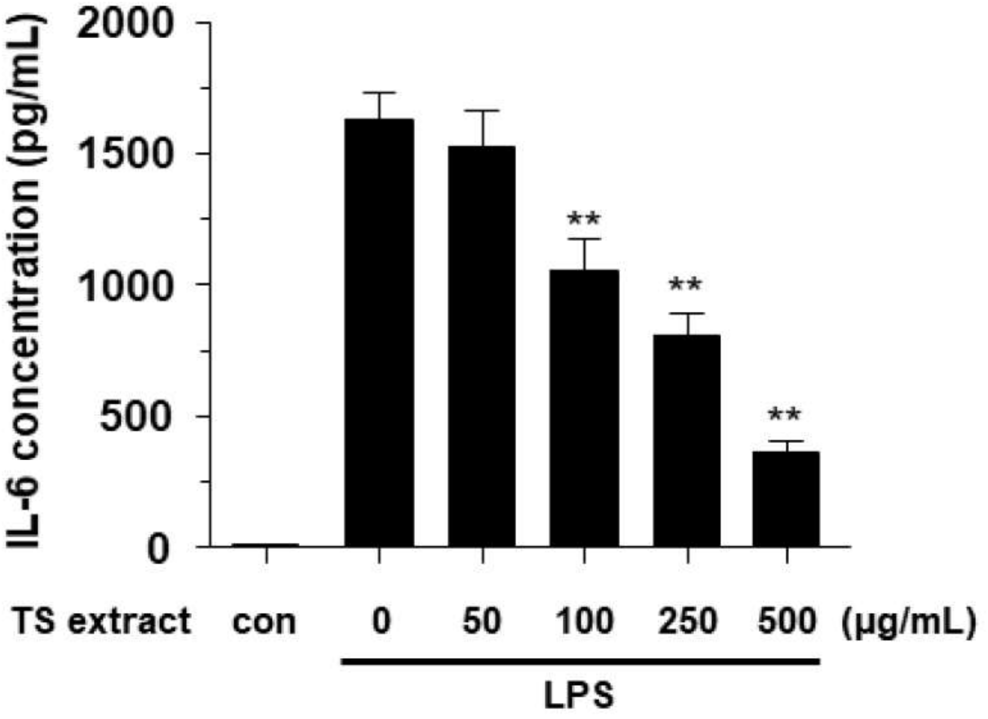

Figure S4. Anti-inflammatory effects of TS extract in LPS-treated RAW264.7 cells. Cells were pre-treated with indicated concentration of TS extract for 1 h and then incubated with 1 ug/mL of LPS for 24 h. Secretion level of IL-6 was determined by ELISA kit. Values are expressed as mean ± SD of three independent experiments; **, $p < 0.001$ versus LPS alone.
